# Effect of dietary supplementation with rumen‐protected amino acids, lysine and methionine, on the performance of Comisana ewes and on the growth of their lambs

**DOI:** 10.1111/asj.70018

**Published:** 2024-12-08

**Authors:** Giulia Grassi, Paola Di Gregorio, Giambattista Capasso, Andrea Rando, Anna Maria Perna

**Affiliations:** ^1^ School of Agricultural, Forestry, Food and Environmental Sciences University of Basilicata Potenza Italy

**Keywords:** blood, Comisana ewes, growth performances of lambs, milk performances, rumen‐protected methionine and lysine

## Abstract

The purpose of the study was to evaluate the effect of supplementing the diets of Comisana ewes with rumen protected methionine and lysine (RP‐ML) for a duration of 60 days on quantitative–qualitative production of milk, antioxidant parameters of milk and blood, biochemical parameters of blood, and lambs' growth performance. Two groups of 15 ewes with equal average body weight were considered for the trial. The control group was administered a standard diet (Control, C) and the experimental group the standard diet supplemented with 1.5% lysine and 1.0% methionine (Treated, T). Results showed that RP‐ML supplementation positively influenced milk yield, fat and protein content, and casein (*p* < 0.01). Furthermore, lambs of the T group, compared with those of the C group, showed a 15% higher growth rate during the suckling period of 42 days. Finally, the total antioxidant capacity of milk, measured by ferric reducing antioxidant power and 2,2′‐azino‐bis(3‐ethylbenzotiazolin‐6‐sulfonic acid assay, was significantly lower in T groups (*p* < 0.05). In conclusion, the results showed that the integration of RP‐ML in ewes could be a valid strategy in order to improve their performances but further investigations are necessary to define the right concentration to be administered to the animals.

## 1 INTRODUCTION

Proteins and their amino acid (AA) components are the most limiting nutrient components for milk yield in ruminants (Park et al., 2020). The protein requirement is linked to the different physiological phases of the animal: growth, pregnancy, and milk yield (Třináctý et al., 2006). Methionine (Met) and lysine (Lys) are considered as the two most limiting AAs involved in milk protein synthesis in dairy ruminants as they constitute the building blocks of casein synthesis (Papadomichelakis et al., 2002; Park et al., 2002) resulting in improved yield of cheese (Junior et al., 2021). Many authors observed that supplementation with Met and Lys can increase milk yield (Giallongo et al., 2016; Papadomichelakis et al., 2002), positively influences the immune response (Lopes et al., 2019; Tsiplakou et al., 2020), oxidative status (Coleman et al., 2020), and reproduction activity (Lopes et al., 2019; Toledo et al., 2017). AAs are also involved in cellular oxidative balance as they participate in the synthesis of taurine and glutathione, determining a positive influence on the antioxidant status of both animals and their products (Naraki et al., 2024). However, a group of North American researchers reported in a meta‐analysis that the nitrogen efficiency in cow milk ranged from 14% to 45%, with a mean of 25%–28% (Huhtanen & Hristov, 2009). Dietary AA can be rapidly degraded by microorganisms in the rumen, and AA from microbial proteins entering the host's small intestine are usually insufficient for high milk yield (Liu et al., 2020). The amount of dietary nitrogen must be well balanced in order to meet microbial requirements (Pathak, 2008). In support, in a study on dairy cows, it was reported the great variability of AA composition of microbial protein that could be deficient in the current content of essential AA required by the animal in production (Sok et al., 2017). Thanks to the scientific contribution, the study and reformulation of animal diets today starts from the qualitative–quantitative understanding of crude proteins, in order to meet the necessary AA requirements in ruminants (Schwab & Broderick, 2017). In fact, scientific research has focused a lot on the importance of a balanced amount of proteins and carbohydrates to synchronize rumen fermentations, thus increasing microbial protein synthesis (Seo et al., 2013). Therefore, it is of fundamental importance to study and regulate protein nutrition with the aim of increasing the efficiency of N use and reducing excess N, that is, the urea content present in milk and feces, which also leads to a significant increase in environmental pollution (Foskolos & Moorby, 2018; Schwab & Broderick, 2017). Previous studies showed that supplementing the feed of lactating ruminants with rumen‐protected methionine consistently increased protein concentration and milk protein yield (Toledo et al., 2017). Furthermore, several studies confirmed that the individual AA transfer efficiency in milk proteins is not constant but decreases with increasing dose (Liu et al., 2020; Liu et al., 2023). Therefore, for improving the production efficiency of dairy animals, the addition of AAs protected from degradation by the rumen is one of the successful strategies (Park et al., 2020). As a consequence, the use of methionine and lysine as supplements in ruminant diets could improve animal performance by affecting feed conversion efficiency and quantity and quality of milk (Khan et al., 2023; Li et al., 2019). Despite the remarkable progress and developments in AA supplementation in animal diets, it is still not common practice to supplement diets with limiting AA. Researchers have investigated the use of rumen‐protected AAs in animal nutrition with encouraging results (Giallongo et al., 2016; Khan et al., 2023; Lee et al., 2015). Although our research is not innovative, it contributes to the understanding of rumen‐protected AA supplementation in small ruminants, in order to reinforce the importance of limiting AA supplementation during production. At present, there is insufficient information on the effect (synergistic or additive) of RP‐AAs on the chemical composition of milk and on the body oxidative stability in ewes. The purpose of the research was to investigate the addition effect of limiting “rumen‐protected” AAs on both quality and quantity of milk produced and the oxidative status of ewes. Furthermore, the growth rate of suckling lambs was evaluated.

## 2 MATERIALS AND METHODS

### 2.1 Experimental animals, management, and study design

This study was carried out in a herd in the province of Potenza (Basilicata, Italy), on a total of 30 multiparous ewes of the Comisana breed; raised in stables; and selected for production level and homogeneous body weight (mean BW 45 ± 3.7 kg). The trial was started immediately after delivery with a conditioning phase (4 days) followed by the experimental phase (56 days) for sampling and analyses. The animals were divided in two groups, managed in two adjacent cages, and controlled for 60 days. The first group (*n* = 15) was administered a control diet (C), formulated using the NDS Professional software (Ver. 3.9.7.11, Rumen Sas, Reggio Emilia, Italy) and using the equations for small ruminants (Cannas et al., 2004); the second group (*n* = 15) was administered the same basic diet with the addition of protected ruminal limiting AAs (T: 1.0% methionine and 1.5% lysine; RP‐ML). The RP‐ML protection was obtained by shielding with fats, resistant to the action of the agents present in the forestomach and incorporated into the concentrated feed. Water was freely available for each group. Fifteen days after birth, the lambs were separated from their mothers and nursed daily for approximately 1 h in the morning and 1 h in the evening, until weaning (42 days of age). The quantity of milk sucked by each lamb was obtained by weighing animals before and after feeding. The BW of the lambs was recorded at birth and at weaning. Blood and milk samples were collected for the determination of the metabolic profile and chemical analyses, every 7 days of the experimental phase. The results of milk and blood analyses were reported as mean values for the entire experimental period. In particular, at the beginning of the experimental period, individual milk samples were collected from each sheep by milking in the parlor. Each day of milk sampling, the sheep were separated from their lambs 2 h before milking, both in the morning and in the afternoon. The milk samples (100 mL of morning milk and 100 mL of evening milk) were then mixed together in a 1:1 ratio into a single sample for chemical analysis. All experimental procedures were approved by the Organismo preposto al Benessere Animale (OpBA) of the University of Basilicata with protocol code OpBA 11_2024_UNIBAS. The composition of the basic ration consisted of red clover hay and concentrated feed. The nutritional parameters and ingredients of the diet administered to the animals are shown in Table 1. The percentage of protein averaged 16%, and the overall raw fiber of the ration was equal to 25%–28% of the total. Rationing was based on the following data: maintenance: 0.58 milk forage unit (MFU/animal) and production: 0.61 MFU/L of milk. The administration of the differentiated ration began immediately after delivery for a total of 64 days.

**TABLE 1:** Chemical composition of the diet.

Parameter	Hay	Concentrate
	**%**	
Dry matter	83.5	12.5
Crude protein	19.0	18.0
Ether extract	3.5	3.0
Crude fiber	32.0	14.0
ADF*	28.7	
NDF**	36.0	
ADL (lignin)***	6.6	
Ash	7.0	10.0
Nitrogen free extract	22.0	
Vit A (UI)		40,000
Vit E (mg)		30.0
Vit D_3_ (UI)		8000

* ADF, acid detergent fiber.

** NDF, neutral detergent fiber.

*** ADL, acid detergent lignin.

### 2.2 Feed chemical composition

Feed chemical composition was determined following the procedures suggested by Mavrommatis et al. (2021). All analytical determinations were done in triplicate.

### 2.3 Milk physicochemical composition

The physicochemical properties of milk from ewes belonging to each group were determined according to Grassi et al. (2022). All analytical determinations (total fat, total protein, ash, sugar, and dry matter) were done in triplicate.

### 2.4 Blood sample collection

Individual blood sampling was carried out in the morning before feed administration. Approximately 10 mL of blood was taken from the jugular vein in heparinized vials with 18‐gauge needle and centrifuged at 4193×*g* for 12 min. Plasma was stored immediately at −20°C until biochemical parameters were determined.

### 2.5 Metabolic profile

In order to determine the metabolic profile from collected blood samples, standard test kits (DiaSys Diagnostic Systems GmbH, Holzheim, Germany) were used for non‐esterified fatty acids (NEFAs), glucose, albumin, total protein, blood urea nitrogen (BUN), cholesterol, and triglycerides. The protocol suggested Iqbal et al. (2022) were followed. A biochemical test analyzer (Star 21 Biochemistry Automatic Analysis; Aspen Diagnostic Pvt. Ltd, Delhi, India) was used to estimate the concentrations of each biochemical parameter.

### 2.6 Antioxidant activity: blood and milk

The 2,2′‐azino‐bis(3‐ethylbenzotiazolin‐6‐sulfonic acid) (ABTS) and ferric reducing antioxidant power (FRAP) tests were performed following the protocol of Tsiplakou et al. (2017) and Benzie and Strain (1996), respectively, on blood samples. The same antioxidant activities were analyzed on the C and T milk samples, following the method suggested by Grassi et al. (2023), with some modifications reported by Tsiplakou et al. (2017). The results were expressed as micromolar of ascorbic acid and percent of inhibition for FRAP and ABTS assay, respectively, on blood and milk samples.

### 2.7 Statistical analysis

The general linear model (GLM) procedure of the SAS software (1996) was used for statistical analysis, and a single‐factor model was applied to evaluate the effect of RP‐AAs added to the diet:

$$y_{i}=\mu +\alpha_{i}+\varepsilon_{j}$$

where *y*_*i*_ are the experimental observations; *μ* is the overall average; *α*_*i*_ is the effect of RP‐AAs (*i*: Lys and Met); *ε*_*j*_ is the residual random error. Arcsine transformation was performed in order to normalize the non‐normal distribution, before analysis (Ahrens et al., 1990). Student's *t* test was used for all comparisons between variables, and differences between means at the 95% confidence level (*p* < 0.05) were considered statistically significant.

## 3 RESULTS AND DISCUSSION

### 3.1 Chemical composition of control and treated milk

Table 2 is shown the average chemical composition of the two groups of sheep, control and treated. By analysis of variance analysis, dietary treatments significantly influenced milk yield, energy, fat, and protein content (*p* < 0.001). In the present study, supplementation of the two AAs, methionine and lysine, increased milk yield in dairy sheep (*p* < 0.01; Table 2). An increase in milk yield (g/day) of approximately 27% in the treated group was observed, going from 944 g/day in the control group to 1288 g/day in the experimental group. Milk protein content (g/day) and the percentage of casein in milk were significantly increased (*p* < 0.01) in ewes fed RP‐ML (Table 2) compared to the control group. It is known that the inclusion in the diet with Met and Lys improves the protein content of milk because they are two limiting AAs for milk protein synthesis (Třináctý et al., 2006). According to in vitro experiments carried out on bovine mammary cells (Nan et al., 2014; Xu et al., 2021), the expression of caseins by mammary epithelial cells is influenced by the relationship between Lys and Met. These data could explain the higher percentage of casein in milk observed in the treated group of the present study. Furthermore, previous studies carried out on Awassi sheep and Shami goats have shown that RP‐AA supplementation positively influenced milk yield and milk protein concentration, which was correlated with a significant increase in casein synthesis (Titi et al., 2022; Yang., 2010). Bertoni and Bertoni (1996) hypothesized that supplementation with RP‐AA in the diet could determine a more balanced AA composition of the absorbable protein with a consequent increase in the quantity of AAs available for absorption in the small intestine, improving the use of proteins for milk secretion. Non‐protein nitrogen (NPN) was significantly higher in the control group (0.37% vs 0.31%; *p* < 0.01). This result is in agreement with the observations of Třináctý et al. (2006) and in contrast with Junior et al. (2021) who observed that the dietary treatment did not influence the value of milk urea nitrogen. In our study, milk fat content was significantly increased in ewes fed RP‐ML, with a content of 42.9 g/day in the control group and 58.9 g/day in the treated group (*p* < 0.01). In agreement with our results, some studies observed that a diet with RP‐methionine increased the fat content in the milk of both sheep (Goulas et al., 2003) and goats (Flores et al., 2009); an increase in fat was also observed in the milk of cows when fed with a pool of Met and Lys (Watanabe et al., 2006; Zanton et al., 2014). Mavrommatis et al. (2021) demonstrated that milk quality increased thanks to RP‐AA supplementation as well as the ratio of the two AAs. Therefore, it is not yet clear why rumen‐protected AA supplementation increases milk fat synthesis. However, AAs in the mammary gland are exclusively used not only for protein synthesis but also for energy production (Rezaei et al., 2016). Indeed, a recent transcriptomic study, carried out on dairy cows, confirms the use of AAs as an energy source for the tricarbonic acid (TCA) cycle (Bionaz et al., 2012). Li et al. (2016) reported the importance of the relationship of essential AAs with the mechanistic target of rapamycin (mTOR) signaling pathways responsible for regulating milk fat synthesis. Furthermore, the same authors put forward the hypothesis that the AA profile could influence the mRNA expression of lipogenic genetic networks and modify the expression of key micro‐RNAs (miRNAs) involved in the control of lipogenic balance. Our results are supported by some authors who investigated the protein content of milk by administering methionine and/or lysine to the animal (Křížová et al., 2014), which have a crucial role in the synthesis of milk proteins (Kudrna et al., 2009) and milk yield. Indeed, Lara et al. (2006) observed in dairy cows that dietary supplementation with methionine resulted in an increase in milk yield and its protein content. Junior et al. (2021) correlated the increase in milk yield in cows fed RP‐Met to a greater efficiency of use of proteinogenic AA absorbed by the mammary gland involved in the synthesis of milk components. However, Třináctý et al. (2006) and Wang et al. (2010) demonstrated that the addition of both rumen‐protected AAs, Lys plus Met, to the diet improved the lactation performance of cows compared to the supplementation with only one rumen‐protected AA. Furthermore, Wang et al. (2010) did not record any interaction between the two AAs, but their effects were additive on lactation performance.

**TABLE 2:** Average milk yield (in grams per day) and chemical composition of milk from ewes fed a diet supplemented with rumen‐protected amino acids (methionine and lysine).

Parameter	Lactation performance				Significance
	Control		Treated		
	*μ* ^1^	*SD* ^2^	*μ*	*SD*	
Milk yield (g/day)	944.00	50.32	1288	60.36	**
Energy (kcal/day)	843.00	42.93	1159	55.32	**
Fat (%)	5.66	0.42	5.85	0.51	
Fat (g/day)	42.90	21.88	58.9	27.09	**
Protein (%)	5.01	0.55	5.55	0.51	
Protein (g/day)	46.90	23.92	65.1	32.55	**
Casein (%)	3.87	0.44	4.31	0.45	**
Whey proteins (%)	0.97	0.11	0.89	0.08	**
NPN (%)	0.37	0.03	0.31	0.03	**
Lactose (%)	4.60	0.31	4.56	0.27	
Ash (%)	0.82	0.07	0.80	0.07	

^1^ *μ* = mean.

^2^ *SD* = standard deviation.

** *p* < 0.01.

### 3.2 Kids growth performance

The BW, growth rate, and efficiency indices of lambs at birth and weaning (42 days) are shown in Table 3. The birth weight of lamb was on average 3.48 ± 0.16 kg, and no significant difference between the control and treated groups was observed. However, in the weaning phase, an average increase in BW of approximately 10.25% was observed in the lambs belonging to the treated group, compared to the control. The growth rate (g/day), during the 42 days of lactation, increased by 15% in the lambs of the RP‐ML group, compared to the control. These results are in agreement with Papadomichelakis et al. (2002) but in contrast with Al‐Qaisi and Titi (2014) who observed no effect of RP‐Met supplementation on the growth rate of kids.

**TABLE 3:** The body weight, growth rate, and efficiency indices of lambs at birth and weaning (42 days) in the control and treated (Met and Lys) groups.

Parameter	Control		Treated		Significance
	*μ* ^1^	*SD* ^2^	*μ*	*SD*	
Birth weight (kg)	3.51	0.21	3.43	0.26	
Weaning weight (kg)	11.47	0.91	12.78	1.02	*
Total LWG (kg)	7.96	0.53	9.35	0.85	*
Growth rate (g/day)	189.52	16.3	222.62	21.64	*
	Efficiency indexes				
Milk/increase (g/g)	6.69	0.94	6.13	0.74	*
Proteins/increase (mg/g)	324	31.76	312	34.32	*
Feed energy/increase (kcal/g)	5.79	0.93	5.50	0.99	*

Abbreviation: LWG = liveweight gain.

^1^ *μ* = mean.

^2^ *SD* = standard deviation.

* *p* < 0.05.

In the present study, production efficiency indices were considered which allow us to evaluate the efficiency of the diet conversion (milk) into an increase in lamb weight (kg; Table 3). On average, to obtain the daily increase of 1 g of BW, a quantity of milk equal to 6.45 g, 320 mg of protein and 5.6 kcal of feed energy were administered to the lambs on the farm. According the comparison between the groups, the T lambs used less milk 8.4% less, approximately 4.0% less protein, and 5.0% less kcal per increase per unit of BW (*p* < 0.05). RP‐ML supplementation may have positively influenced the biological value of proteins, because per unit increase in lambs, the lambs of the treated ewes consumed approximately 4% less than the lambs of the control group. In line with our results, Titi (2017) highlighted that the milk/gain ratio (milk fed during 60 days: BW gain, kg/kg) was better for kids of goats fed RP‐Met.

### 3.3 Effects of RP‐ML supplementation on plasma biochemical parameters

In this study, the analysis of blood samples was carried out to evaluate the health and nutritional status of the animals (Table 4). No significant differences were detected between the two groups at the level of plasma concentrations of glucose, NEFA, urea nitrogen, and total triglycerides (*p* > 0.05; Table 4). The total cholesterol content, in contrast, was significantly lower in the T group compared to the C (*p* < 0.01). Although triglyceride contents were not significantly altered (*p* > 0.05), they were numerically decreased with treatment. Zhang et al. (2013) explained that the decrease in blood concentrations of triglycerides and cholesterol could be due to the increase in fat mobilization, which could be favored by AA due to their role as methyl donors (Davidson et al., 2008). Li et al. (2022) also studied the biochemical parameters of blood plasma in goats fed RP‐Met, although they found no significant differences for total cholesterol.

**TABLE 4:** Total concentrations of glucose, albumin, proteins, NEFA, urea nitrogen, and total triglycerides in blood plasma samples of control and treated ewe groups.

Parameter	Control		Treated		Significance
	*μ* ^1^	*SD* ^2^	*μ*	*SD*	
Glucose (mmol/L)	2.56	0.05	2.63	0.06	
Triglycerides (mmol/L)	0.15	0.01	0.13	0.01	
Cholesterol (mmol/L)	1.36	0.06	1.19	0.05	**
Proteins (g/L)	73.96	4.46	72.16	6.04	
Albumin (g/L)	30.29	2.12	31.71	1.27	
Urea (mmol/L)	5.84	0.19	5.92	0.29	
NEFA (μmol/L)	707.53	53.77	749.63	81.29	

Abbreviation: NEFA, non‐esterified fatty acid.

^1^ *μ* = mean.

^2^ *SD* = standard deviation.

** *p* < 0.01.

### 3.4 Antioxidant activity in plasma and milk

In the present study, the free radical scavenging tests, ABTS and FRAP, were used for the determination of total antioxidant content (TAC) in plasma and milk (Table 5).

**TABLE 5:** Antioxidant activity (FRAP and ABTS assays) in plasma and milk samples of both control and treated ewes' groups.

Parameter	Control		Treated		Significance
	*μ* ^1^	*SD* ^2^	*μ*	*SD*	
	Plasma				
FRAP (μMol ascorbic acid)	3.42	0.23	3.15	0.16	
ABTS (I %)	34.82	1.64	34.19	1.76	
	Milk				
FRAP (μMol ascorbic acid)	5.22	0.28	4.54	0.17	*
ABTS (I %)	30.14	2.38	27.62	1.83	*

Abbreviations: ABTS, 2,2′‐azino‐bis(3‐ethylbenzotiazolin‐6‐sulfonic acid); FRAP, ferric reducing antioxidant power; I %, inhibition percentage.

^1^ *μ* = mean;

^2^ *SD* = standard deviation.

* *p* < 0.05.

The complexity of a biological sample and the heterogeneity of antioxidant chemistry prevent quantification of the antioxidant components present in the sample. Therefore, it is more significant to determine the total antioxidant capacity (TAC) in order to quantify the antioxidant content present in a sample. The ABTS and FRAP assays were used in this study to measure TAC in plasma and milk to evaluate all synergistic and cumulative interactions between known and unknown compounds (Fraga et al., 2014). In blood plasma, RP‐ML supplementation did not significantly affect antioxidant activity. On average, the FRAP value was 3.3 μM ascorbic acid and the inhibition percentage (I%) was 34.5%, with no significant differences between the two groups.

Therefore, it is good to consider the added quantities and the synergistic effects given by the ratio of AA in animal diets both to obtain the optimal inclusion levels but also to avoid pro‐oxidant effects, given by a strong physiological imbalance. The total antioxidant capacity of milk, measured by FRAP and ABTS tests, was significantly lower in group T than in group C (*p* < 0.05). In fact, for the FRAP assay, the values of the RP‐ML group were 13% lower than the control, while for the ABTS assay, the reduction was more limited, equal to 8.4%. These results are in line with those observed by Mavrommatis et al. (2021) in Chios sheep fed with a rumen‐protected Met and Lys supplement. Indeed, dietary supplementation with such AAs simultaneously reduced the total antioxidant capacity of milk. Although the role of methionine and lysine, as antioxidant compounds, has been studied extensively in ruminants (Khan et al., 2023), the decrease in antioxidant activity observed in our study is difficult to explain and is not supported in the literature. Furthermore, both the concentration and the mechanism of action of both essential AAs that are involved in cell detoxification could have a negative effect on the antioxidant status of milk (Mavrommatis et al., 2021). Further data are necessary to explain this result. We can hypothesize that a higher concentration of rumen‐protected AA in treated milk could favor the formation of complexes with other macromolecules such as phenols or react with other components and form disulfide bridges. The possible formation of complexes, therefore, would not make their antioxidant power available, determined by ABTS and FRAP assays.

## CONFLICT OF INTEREST STATEMENT

The authors declare no conflicts of interest.

## Data Availability

Data will be made available on request.
